# Cost of Chiari I Malformation Surgery: Comparison of Treatment at Children’s Hospitals Versus Non-children’s Hospitals

**DOI:** 10.7759/cureus.12866

**Published:** 2021-01-22

**Authors:** Jessica Lane, Amber L Schilling, Christopher Hollenbeak, Elias Rizk

**Affiliations:** 1 Neurosurgery, Penn State Health Milton S. Hershey Medical Center, Hershey, USA; 2 Surgery, Penn State Health Milton S. Hershey Medical Center, Hershey, USA; 3 Public Health, Penn State Health Milton S. Hershey Medical Center, Hershey, USA

**Keywords:** cost, length of stay, kids' inpatient database, chiari i malformation

## Abstract

Chiari I malformation is a common entity in pediatric neurosurgery. Prior studies have shown that surgical treatment at children’s hospitals (CH) is associated with higher costs compared to non-children’s hospitals (NCH) for other diagnoses. Therefore, we hypothesized that costs would be increased for the treatment of Chiari I malformation at a CH. Data were extracted from the Agency for Healthcare Research and Quality’s (AHRQ) Healthcare Cost and Utilization Project (HCUP) Kids' Inpatient Database (KID). Patients who underwent surgery for Chiari I malformation were identified using International Classification of Diseases, 9th Edition, Clinical Modification (ICD-9-CM) diagnosis and procedure codes. Univariate statistical tests, multivariable linear regression models, and propensity score matching were utilized to determine differences in hospital length of stay (LOS) and costs between patients treated at CH versus NCH.

Treatment at a CH was associated with significantly higher costs compared to treatment at an NCH while hospital LOS and mortality were similar. In the multivariable linear regression model, the adjusted average cost for surgical treatment of Chiari I malformation was $13,716, and treatment at a CH was associated with an additional $6,343 (p<0.0001). Similar results were seen after propensity score matching: costs for treatment at a CH were $6,047 higher than they were for treatment at an NCH (p<0.0001). In our analysis, a significant increase in cost was seen with treatment at a CH while controlling for patient demographics and hospital characteristics, as well as imbalanced covariates between the cohorts. Further investigation is warranted to determine the drivers of increased cost outside of the patient and hospital characteristics we analyzed in our study.

## Introduction

Chiari I malformation is a common pediatric neurosurgical issue. Although the prevalence of Chiari I malformation is difficult to estimate, epidemiological studies suggest that it occurs in 0.7% to 3.6% of the U.S. population [1-3]. The surgical treatment rate has been estimated at 2.5 per 100,000 patient-years in patients under 20 years old [4]. Unadjusted cost estimates for this surgery range from US$7,000 to US$30,000 for a single admission [5]. Surgery for Chiari I malformation is performed with considerable frequency at both designated Childrens’ Hospitals (CH) and Non-childrens’ Hospitals (NCH) which allows for comparison of these two populations. 

As healthcare-related expenditures continue to grow, there is an increasing emphasis on evaluating drivers of increased cost and the added value it yields [6]. Within pediatrics, and specifically, in pediatric surgical specialties, recent attention has been given to the differences in costs and outcomes for children receiving surgical care at a freestanding CH versus other facilities. For a number of surgical and nonsurgical diagnoses, studies using national samples have yielded evidence that treatment in a free-standing CH is associated with higher costs relative to treatment at other hospitals [6-11]. Increased costs may be associated with more complex or challenging patients and may or may not lead to improved outcomes. However, this has not been fully elucidated in the literature. The goal of this study was to compare the cost of patients undergoing surgery for Chiari I malformation at CHs versus NCHs using a national sample and controlling for potentially confounding variables and selection bias due to imbalanced covariates.

## Materials and methods

Data for this study were extracted from the Agency for Healthcare Research and Quality’s (AHRQ) Healthcare Cost and Utilization Project (HCUP) Kids' Inpatient Database (KID). HCUP-KID is a national, all-payer sample of pediatric inpatient discharge data. Data from HCUP-KID have been released every three years, starting with the year 1997. Due to the change in medical coding from International Classification of Diseases, 9th Edition, Clinical Modification (ICD-9-CM) to ICD-10-CM that took place late in the year 2015, our analysis was limited to years which utilized ICD-9-CM coding only, including admissions during the years 2003, 2006, 2009, and 2012.

The patient cohort was identified by a primary or secondary ICD-9-CM diagnosis code of 348.4 (compression of brain) combined with an ICD-9-CM procedure code of 01.24 (other craniotomy) or 03.09 (other exploration and decompression of spinal canal) which has been previously validated [12]. Admission to a CH was defined using the National Association of Children’s Hospitals and Related Institutions (NACHRI) classification system. Hospitals that were classified as a Children’s General Hospital or a Children’s Specialty Hospital were considered a CH; hospitals categorized as having only a children’s unit within the larger general hospital were not considered a designated CH and were instead classified as an NCH.

Outcomes examined in this study were the length of hospital stay (LOS) and total hospital admission costs. Costs were estimated from the perspective of the provider using cost-to-charge ratios. In addition, all costs were inflated to 2016 USD using the Medical Care Component of the Consumer Price Index (CPI) of the United States Bureau of Labor Statistics (BLS) [13].

The goal of the statistical analysis was to determine whether patients with Chiari I malformation treated at CHs had significant differences in LOS and costs compared to patients treated at NCHs. Univariate statistical analysis included chi-square tests for comparisons of categorical variables between CH and NCH and t-tests for comparisons of continuous variables. Multivariable analysis controlling for potential confounders was performed using linear regression for LOS and costs.

In order to control for covariate imbalance between patients treated at CHs versus patients treated at NCHs, a propensity score analysis was undertaken where patients treated at CHs were matched 1:1 without replacement using a nearest neighbor approach and a caliper restriction of 0.2 standard deviations. The outcome measure for the propensity score analysis was the average effect of treatment on the treated (ATT), which represents the estimated difference in outcome between patients treated at CHs versus the expected outcome had those patients been treated at NCHs. Bootstrapping with 500 replicates was performed to estimate the 95% confidence intervals for the ATTs and to compute p-values. 

The threshold for statistical significance was set at p<0.05, and all statistical analyses were performed using Stata, version 15 (College Station, Texas, USA).

## Results

Characteristics of patients included in the analysis, stratified by type of hospital where care was received, are presented in Table 1. 

**Table 1:** Demographic characteristics of the Chiari cohort *Notes: Other race/ethnicity category includes native American, unknown race, and racial or ethnic groups not otherwise categorized by the Healthcare Cost and Utilization Project Other payer category includes no charge and patients whose payers were unknown The number of beds for small, medium, and large is determined by region, location, and teaching status; refer to the HCUP-KID codebook for additional information (https://www.hcup-us.ahrq.gov/db/vars/hosp_bedsize/kidnote.jsp.) Other admission type includes trauma and patients whose admission types were unknown APR-DRG, All Patient Refined Diagnosis Related Group LOS, Length of stay Costs were inflated to 2016 US dollars using the medical care component of the Bureau of Labor Statistics Consumer Price Index

	Children's	Non-children's	
	hospital	hospital	
Variable	(N=1603)	(N=2675)	P-value
Age (years, mean)	9.1	11.3	<0.0001
0-5	29.8%	21.8%	
6-11	31.3%	23.9%	
12-15	24.3%	23.1%	
≥16	14.7%	31.1%	
Race/ethnicity			<0.0001
White	54.7%	62.1%	
Black	4.5%	6.5%	
Hispanic	9.6%	6.3%	
Asian	1.8%	1.9%	
Other*	29.4%	23.3%	
Sex			0.053
Female	53.3%	56.4%	
Male	46.7%	43.6%	
Payer type			<0.0001
Medicaid	33.6%	27.3%	
Commercial	58.3%	64.9%	
Self-pay	2.0%	1.2%	
Other*	6.1%	6.6%	
Hospital bedsize*			<0.0001
Small	25.8%	1.1%	
Medium	46.8%	7.6%	
Large	18.3%	89.3%	
Unknown*	9.2%	2.1%	
Hospital region			<0.0001
Northeast	11.2%	18.1%	
Midwest	34.4%	29.7%	
South	28.6%	37.3%	
West	25.9%	14.8%	
Admission type			<0.0001
Emergent	2.7%	2.1%	
Urgent	5.7%	3.7%	
Elective	48.1%	54.4%	
Other*	43.5%	39.7%	
APR-DRG severity risk	1.7	1.6	0.004
0 (no class specified)	0.0%	0.0%	
1 (minor loss of function)	39.2%	43.7%	
2 (moderate loss of function)	51.3%	48.9%	
3-4 (major or extreme loss of function)	9.5%	7.5%	
Year			0.074
2003	15.7%	13.9%	
2006	20.8%	22.1%	
2009	24.6%	27.3%	
2012	39.0%	36.8%	
LOS (days)	4.1	3.9	0.0572
Cost (2016 US dollars)*	$23,131.61	$16,535.58	<0.0001
Mortality	0.19%	0.04%	0.121

Children treated at CHs were significantly younger compared to patients treated at NCHs (mean age of 9.1 years vs 11.3 years; p<0.0001), with the most notable difference noted among patients in the ≥16 age category (14.7% of patients in the CH group vs 31.1% of patients in the NCH group). The patients in both cohorts were predominately white, with a slightly higher percentage of black patients represented in the NCH group (4.5% in CH vs 6.5% in NCH), but an overall higher percentage of non-white patients in the CH group (45.3% in CH vs 37.9% in NCH). 

The difference in the distribution of payer type between the two cohorts was statistically significant, with a higher percentage of patients covered by Medicaid treated at CHs (33.6% in CH vs 27.3% in NCH) and a higher percentage of patients covered by commercial insurance treated at NCHs (64.9% in NCH vs 58.3% in CH). Nearly 90% of NCH patients were treated at large hospitals, and more than 70% of CH patients were treated at small- and medium-sized hospitals. Elective cases had higher representation in the NCH cohort (54.4% in NCH vs 48.1% in CH), while urgent cases had higher representation in the CH group (5.7% in CH vs 3.7% in NCH). Although the difference in the All Patients Refined Diagnosis Related Groups (APR-DRG) severity risk scores was statistically significant (p=0.004), the absolute difference between means was small (1.7 in CH vs 1.6 in NCH).

The difference in unadjusted hospital length of stay (LOS) between CHs and NCHs was not statistically significant (4.1 days for CH vs 3.9 days for NCH; p=0.0572). However, unadjusted costs were significantly higher for patients in the CH group ($23,131 versus $16,535; p<0.0001). Overall, mortality rates were very low and the difference between groups was not statistically significant (0.19% in CH vs 0.04% in NCH; p=0.121).

**Figure 1:**
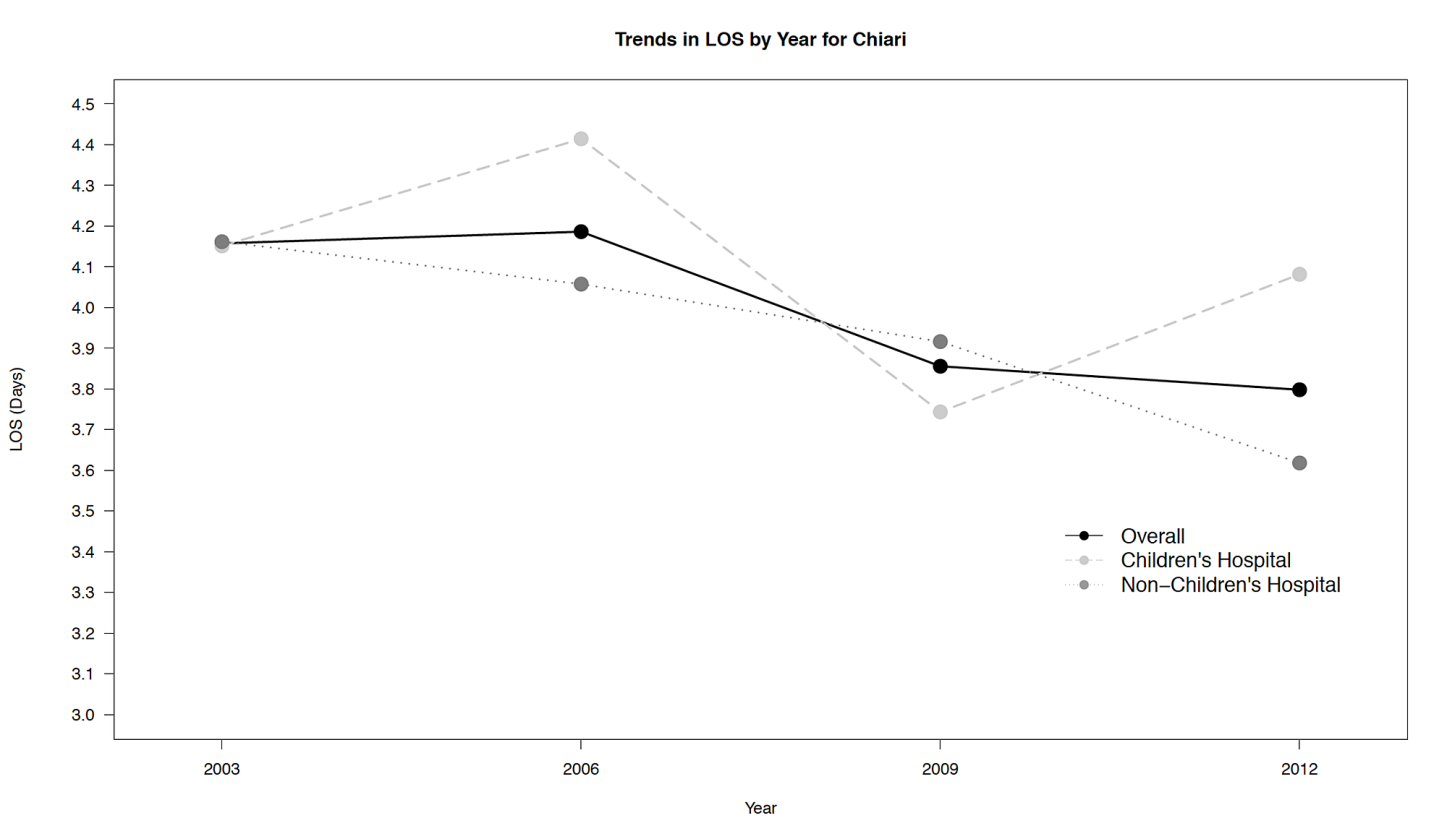
Trends in length of stay (unadjusted) over time LOS, length of stay

Figure 1 presents time trends in unadjusted, average LOS for CHs and NCHs from 2003 to 2012 and shows that the average LOS has fallen slightly over the study period.

**Figure 2:**
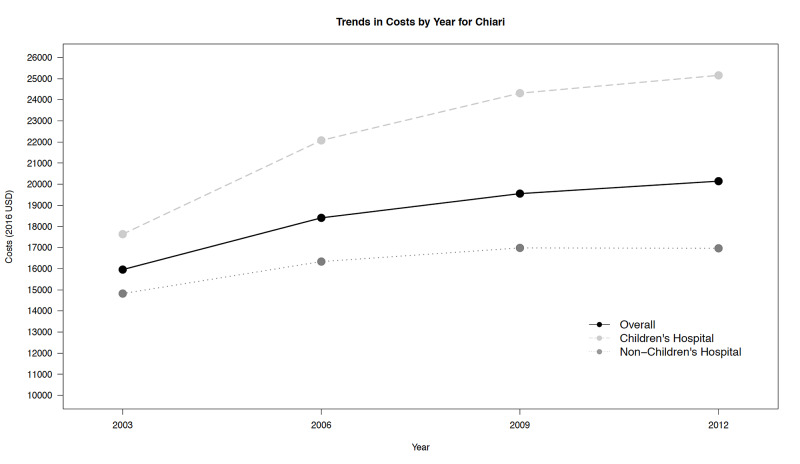
Trends in costs (unadjusted) over time

It also suggests that LOS for this procedure is similar between hospital types. Trends in unadjusted costs are presented in Figure 2, and unlike LOS, have increased for both hospital types over time, with higher costs consistently associated with CHs. In addition, the magnitude of the cost growth trend for CHs was significantly higher relative to the growth trend observed for NCHs.

The linear regression model for LOS is provided in Table 2.

**Table 2:** Results of the linear regression for length of stay R^2^=0.1332 LOS, Length of stay

		95% Confidence		
Covariate	Coefficient	Lower	Upper	P-value
Age				
0-5	0.1180	-0.1536	0.3896	0.3940
6-11	REFERENCE			
12-15	0.0697	-0.2047	0.3441	0.6190
≥16	0.3709	0.0937	0.6480	0.0090
Race/ethnicity				
White	REFERENCE			
Black	0.2929	-0.1363	0.7220	0.1810
Hispanic	0.4567	0.0685	0.8449	0.0210
Asian	0.3778	-0.3530	1.1086	0.3110
Other	-0.0654	-0.3144	0.1836	0.6070
Sex				
Female	0.2750	0.0783	0.4717	0.0060
Male	REFERENCE			
Payer type				
Medicaid	0.0872	-0.1384	0.3128	0.4490
Commercial	REFERENCE			
Self-pay	-0.6386	-1.4528	0.1757	0.1240
Other	0.1136	-0.2927	0.5200	0.5840
Hospital bedsize				
Small	-0.3109	-0.7294	0.1075	0.1450
Medium	-0.2456	-0.5628	0.0717	0.1290
Large	REFERENCE			
Unknown	-0.1146	-0.6379	0.4086	0.6680
Hospital region				
Northeast	0.1099	-0.1923	0.4120	0.4760
Midwest	0.3902	0.1397	0.6407	0.0020
South	REFERENCE			
West	0.1037	-0.2030	0.4103	0.5080
Admission type				
Emergent	2.2496	1.5936	2.9057	<0.0001
Urgent	0.8239	0.3399	1.3079	0.0010
Elective	REFERENCE			
Other	0.6169	0.0440	1.1899	0.0350
APR-DRG severity risk				
1 (minor loss of function)	-0.3393	-0.5440	-0.1346	0.0010
2 (moderate loss of function)	REFERENCE			
3-4 (major or extreme loss of function)	3.9133	3.5482	4.2785	<0.0001
Year				
2003	0.9015	0.2801	1.5230	0.0040
2006	0.9115	0.3127	1.5103	0.0030
2009	0.5161	-0.0835	1.1157	0.0920
2012	REFERENCE			
Children's hospital	0.2808	-0.0221	0.5836	0.0690
Constant (LOS, in days, for reference patient)	2.4351	1.7855	3.0846	<0.0001

After controlling for potential confounders, treatment at a CH was not significantly associated with LOS. However, higher APR-DRG severity score and greater surgical urgency were associated with significantly longer LOS (3.9-day increase for APR-DRG severity score of 3-4 compared to APR-DRG severity score of 2 and a 2.2-day increase for emergent cases compared to elective cases; p<0.0001 for both comparisons). The results of the linear regression for cost are provided in Table 3.

**Table 3:** Results of the linear regression for costs R^2^=0.1974

		95% Confidence		
Covariate	Coefficient	Lower	Upper	P-value
Age				
0-5	-$606.28	-$1,670.27	$457.71	0.2640
6-11	REFERENCE			
12-15	$4.71	-$1,070.07	$1,079.50	0.9930
≥16	$649.61	-$435.94	$1,735.16	0.2410
Race/ethnicity				
White	REFERENCE			
Black	$677.00	-$1,004.00	$2,358.01	0.4300
Hispanic	$3,182.51	$1,662.01	$4,703.02	<0.0001
Asian	$1,691.12	-$1,171.47	$4,553.71	0.2470
Other	$978.33	$2.94	$1,953.72	0.0490
Sex				
Female	$312.37	-$458.15	$1,082.90	0.4270
Male	REFERENCE			
Payer type				
Medicaid	-$810.33	-$1,694.07	$73.41	0.0720
Commercial	REFERENCE			
Self-pay	-$1,489.93	-$4,679.57	$1,699.71	0.3600
Other	$217.92	-$1,373.70	$1,809.54	0.7880
Hospital bedsize				
Small	$175.46	-$1,463.56	$1,814.48	0.8340
Medium	-$1,600.68	-$2,843.45	-$357.91	0.0120
Large	REFERENCE			
Unknown	$433.62	-$1,615.98	$2,483.22	0.6780
Hospital region				
Northeast	$2,007.28	$823.73	$3,190.83	0.0010
Midwest	$2,840.45	$1,859.23	$3,821.67	<0.0001
South	REFERENCE			
West	$4,382.27	$3,181.12	$5,583.43	<0.0001
Admission type				
Emergent	$4,186.61	$1,616.80	$6,756.42	0.0010
Urgent	-$620.77	-$2,516.64	$1,275.10	0.5210
Elective	REFERENCE			
Other	$1,443.28	-$800.98	$3,687.54	0.2070
APR-DRG severity risk				
1 (minor loss of function)	-$1,848.47	-$2,650.27	-$1,046.67	<0.0001
2 (moderate loss of function)	REFERENCE			
3-4 (major or etreme loss of function)	$15,679.19	$14,248.84	$17,109.55	<0.0001
Year				
2003	-$3,417.90	-$5,852.27	-$983.53	0.0060
2006	-$539.90	-$2,885.29	$1,805.49	0.6520
2009	$502.53	-$1,846.07	$2,851.12	0.6750
2012	REFERENCE			
Children's hospital	$6,343.32	$5,157.04	$7,529.60	<0.0001
Constant (cost for reference patient)	$13,715.66	$11,171.38	$16,259.93	<0.0001

CHs were associated with significantly higher costs compared to NCHs, with an incremental increase of $6,343 per case (p<0.0001) above the adjusted mean of $13,716. Higher APR-DRG severity score and greater surgical urgency were also associated with significantly higher costs in this model (increase of $15,679 for APR-DRG score of 3-4 compared to APR-DRG score of 2 and an increase of $4,187 for emergent cases compared to elective cases; p<0.0001 and p=0.001, respectively).

In order to control for the covariate imbalance between patients treated at CH and NCH observed in Table 1, we used a propensity score matching analysis to validate the multivariable model, the results of which are shown in Table 4. 

**Table 4:** Results of the propensity score matching analysis for length of stay and costs ATT, Average effect of treatment on the treated LOS, Length of stay

	Children's	Non-children's		95% Confidence		
Variable	Hospital	Hospital	ATT	Lower	Upper	P-value
LOS (days)	4.21	3.95	0.26	-0.27	0.78	0.338
Cost (2016 USD)	$23,781.15	$17,733.73	$6,047.42	$4,051.49	$8,043.35	<0.0001

CH was associated with an insignificant increase in LOS of 0.26 days (p=0.338), but a statistically significant increase in costs of $6,047 (p<0.0001), which suggests robustness in our multivariable analyses.

## Discussion

This study used a large, national cohort from HCUP-KID to examine the differences in LOS and cost for surgical treatment of Chiari I malformation at CHs vs NCHs. In terms of demographic breakdown, average age was younger at CH than at NCH and there was more representation of the oldest age cohort at NCH. This may represent overall admitting trends by hospital type, and while age was found to be associated with LOS, it was not associated with a difference in cost. Hospital size was also significantly different between the two groups. Larger NCH may benefit from the economics of higher volumes, although they do not necessarily perform a greater number of these specific procedures on this patient population. Additionally, there was a regional difference in the cost of care with hospitals in the West having the highest average cost. While all of these factors were significant in the linear regression model, they did not reduce the ATT in the propensity score analysis.

We found that although LOS was similar between cohorts, the cost of treatment was significantly higher at CHs compared to NCHs. Findings from univariate, multivariable linear regression, and propensity score matching analyses each corroborated this conclusion. This finding is consistent with previous studies that have made cost comparisons between CH and NCH for other surgical diagnoses using different comparison methods. Additionally, this large cohort uses four different time points (years 2003, 2006, 2009, and 2012) from the HCUP-KID to examine trends over time, which suggested that the cost of surgery for Chiari I malformation is increasing over time and the trend is increasing at a significantly higher rate among CH.

Propensity score analysis has been used more frequently in recent years when a randomized controlled trial is not feasible. It is one way to account for selection bias between cohorts when patients in one cohort may be inherently different or have a different distribution of covariates than those in another. In our cohort, APR-DRG severity score, which was our major marker for patient complexity and risk, was significantly associated with both cost and LOS in our linear regression model, as was the urgency of the surgical case. Since designated CHs may inherently treat more severe and urgent cases, propensity score matching helped to neutralize this potential bias. Our analysis showed a significant treatment effect (or cost increase) similar to that predicted by the linear regression model, strengthening the conclusion that hospital type was a key driver of increased costs.

Our study has several important limitations. Outcomes, such as readmissions, patient satisfaction scores, and others, may be relevant in discussions of cost and value-based care but were not addressed in our study because they are not available in HCUP-KID. The APR-DRG severity score was used as our marker for illness severity, expected mortality, and resource utilization because it was readily available in the dataset. However, this marker is likely an oversimplification of case complexity in pediatric patients with Chiari I malformation.

Finally, this study was not designed to address differing surgical practices or approaches between surgeons and/or across regions and hospital types, factors that surely affect cost and outcomes. As current research continues to investigate the best operative methods for treating Chiari I malformation, practices between surgeons and across hospital types may become more uniform and create less of a cost differential.

## Conclusions

This study demonstrated that for a large national cohort, the cost of surgical treatment for pediatric Chiari I malformation patients was higher at CHs when compared to NCHs. While this cost differential has been suggested to stem from differences in the patient populations admitted to CHs versus NCHs, an incremental cost increase of $6,343 was seen with patients treated at CHs in our multivariable model. This was validated in our propensity score matching analyses. Finally, we have demonstrated that the trend of increased cost associated with CH treatment is strengthening over time. Taken together, these findings demonstrate an increasing cost differential for treatment of the same condition. While, as outlined in the discussion, there are significant limitations to our analysis especially concerning the variable complexity of disease presentation and treatment, further research should investigate the specific drivers of increased cost with CH treatment.
